# 

**Published:** 1858-09

**Authors:** 


### INDEX TO THE FOURTH VOLUME

OF THE

### ATLANTA MEDICAL & SURGICAL JOURNAL.

PAGE.

### A

Arsenic in Cutaneous Diseases, by Jno, R. Cushing, M, D., South Butler, Ala.,......... 1
American Medical Association,.................. 45
Atlanta Medical College—Close of the Session,.............................................. 50
Advertisements........................................ 64
Annual Address to Graduates A. M. C., at the Commencement, Sept. 2d, 1858, by C, B. Nottingham, M. D., of Macon,............ 65
Atlanta Medical College,........................ 119
Anaesthesia and Electricity,..................... 167
Another Year,........................................ 311
Atlanta Medical College Clinic, November and December, 1858,.............................. 325
Atlanta Medical College Museum,............ 377
Astonishing Impudence,........................... 431
A Grain of Wheat from a Bushel of Chaff, 435
Atlanta Medical College,......................... 443
Atlanta Medical College Clinic,................. 473
Antrum or Maxillary Sinus, Diseases of—by F. D. Thurman, Dentist, Atlanta, Ga.,..... 478
American Medical Association,.................. 505
Abortion, Criminal—Cases illustration of... 553
Antrum or Maxillary Sinus—Diseases of, By F. D. Thurmond, Dentist, Atlanta, Ga. 594
American Medical Association,................... 629
Atlanta Medical College,........................... 638
Atlanta Medical College Museum,.............. 640
Address: Introductory to the Fifth Course of Lectures in the Atlanta Medical College, by Jos. P. Logan, M. D., Professor of Physiology and Diseases of Women and Children, Atlanta, Ga.,.......................... 645
An Original Number,............................... 763
Aconite—Remarks upon the Therapeutical Action of—by J. R. Cushing, M. D., South Butler, Ala.,............................................. 691

### B

Bibliographical,...................................... 256
Bandaging Tight—Ill Effects of,................ 258
Blood-letting Question in Olden Time,........ 275
Bibliographical,...................................... 311
do. ...................................... 55
Boring, Dr............................................ 377
Bright, Dr.—Death of,.............................. 387
Bibliographical,...................................... 765
Bathing Imprudently—Cases of Sudden Death attributed to,................................ 501
"Bellevue Hospital," Richmond, Va., Reports of Cases occurring in, by Thomas Pollard, M. D., one of the Physicians to the Hospital.......................................... 725
"Bibron's Antidote," by D. O. C. Heery, M. D., Atlanta, Ga.,................................... 732

### C

Child-Bed Fever, An Essay, Read before the Medical Association of the State of Georgia, by S. H. Dean, M. D., of Conyers, Georgia,.............................................. 719
College Announcements,........................... 127
Cataract, Operations for,........................... 224
Cyanosis Neonatorum, by A. G. Thomas, A. M., M. D., Atlanta, Ga.,...................... 268
Convulsions, Infantile,.............................. 305
Chloroform in Infantile Convulsions,........ 317
Cinchonia in Menorrhagia—Case, by Robert Battey, M. D., Rome, Ga.,.......................... 734
Coroner's Inquest,.................................... 322
College Announcements, Received,............ 63
Cholera Infantum—Post Mortem Appearances of, with Cases,............................... 351
Convulsions in Children treated with large doses of Sulphate of Quinine, by E, H. W. Hunter, M. D., Louisville, Ga.,................ 367
Clinical Reports,...................................... 377
Cataract, Essay on.................................... 379
Charlatanry, Empiricism, Quackery,.......... 384
Cases from the Note Book of J. R. Cushing, M. D., of South Butler, Ala.,................. 394
Chloroform in Labor, by Jos. P. Logan, M. D., Atlanta, Ga.......................................... 398
College Clinic, by J. G. Westmoreland, M. D., Atlanta, Ga.,........................................ 405
Charleston Medical College,...................... 509
Chinchonia—Sulphate of, by Robt. Battey, M. D,, Rome, Ga.,.................................. 531
Calomel—Remarks on Modus Operandi of, by Hayden Coe, M. D., Atlanta, Ga..........533
Croupal Diphtheria, by F. H. Evans, M. D., of Aberdeen, Mississippi,.......................... 738
Cancer—Treatment of—Cancer Quacks,..... 577
Convention of Medical Teachers,.............. 632
Contributors,.......................................... 638
Correspondence—Extract from,................ 635
Convention of Medical Teachers,.............. 640

### D

Deafness—Congenital, Electricity in......... 257
Deal with the Druggist,........................... 385
Dead, Alive............................................ 492
Discutient Application to the Indurated Epididymis,.......................................... 515
Dentition, by J. P. H. Brown, Dentist, Atlanta, Ga.,............................................ 740

### E

Endemic and Indigenous Diseases,............ 292
Epigenesis and Sterility, practical remarks on ....................................................... 37
Essay, read before the Atlanta Medical Society, by Hayden Coe, M. D., Atlanta, Ga. 389
Extracts from Private Correspondence,..... 444
Errata,................................................... 451
Epilepsy—A Case Treated by Tracheotomy 493
Epilepsy for thirty-two years in a man, aged 44, with discoloration of the skin from Nitrate of Silver—Operation of Castration,.............................................. 501
Excision of the Knee Joint—Statistics on, 512

### F

Foreign Correspondence, by Wm. J. Arrington, M. D., Dublin, Ireland,............... 193

Food—Histogenetic, and Non-Histogenetic, by A. G. Thomas, A. M., M. D., Atlanta, 197

French Army Surgeons—Rewards of,......... 321

Fort Pitt Hospital, Reports of Cases of,....... 497

Fort, Dr. Tomlinson Death of,.................. 639

### G

Georgia Medical Society,.......................... 375
Green, Dr. and the New York Academy of Medicine,.......................................... 382
Groux, Eugene A.—"Congenital Fissure of the Sternum,"........................................ 446

PAGE.
Griffin Auxilliary Medical Society,............ 508
Galvanic Anæsthesia,.............................. 512

H

Hydrocephalus, Subject of, Phenominal History of, and Post-Mortem Measurements, &c., with Remarks upon the Etiology of, by Jno. T. Banks, M. D., of Zebulon, Georgia,.............................................. 81
Heart Sounds, Clinical Study of,............... 88
Hare, M.D., Professor Robert, Biographical Sketch of,............................................ 115
Hemorrhages, Sulphate of Zinc in, by Wm. Hauser, M. D.. Spier's Turn-Out, Ga.,....... 135
Hygiene of Costume,.............................. 2[illegible]2
Hospital, St. George's.............................. 252
Hemorrhage from Extracting Teeth, by F. D. Thurmond, Dentist. Atlanta, Ga.,....... 7
Humulus Lupulus,.................................. 386
Hydrophobia from the Bite of a Pole-Cat,.. 386
Heart Sounds, Clinical Study of,............... 481

I

Itch, Treatment of,................................. 515
Inversion of the Womb, by Walter Channing, M. D.,........................................... 769

J

Joints, Injuries and Diseases of, as Illustrated,.................................................. 237
Journal, Receipts for............................... 260

K

Kameela, The New Anthelmintic...............563
Kentucky Medical Society, Report of the Committee on Suits for Mal-Practice, Submitted at the Fourth Annual Meeting, 620

L

Lee's Dr. Reclamation for Dr. Paine,......... 97
Lying-In Hospital, Dublin, Oct. 11th, 1858,... 357
Lives without Eating, The woman who..... 490
Lives without Eating, The woman who.... 507
Local Anæsthesia,................................... 513
London Correspondence,.......................... 584
Liver, Gun-shot wound of, Recovery, by E. M. Pendleton, M. D., of Sparta, Ga.,........ 589
List of Payments,.................................... 771

M

Medical Ethics, a point in,........................ 112
McCaw, M. D., Prof. Jas. B........................ 120
Medicine, Primary School of..................... 128
Medical Science, Progress of—Selections from Journals on the Treatment of Croup 163
Medicine, Rational, a Review,................... 178
Medical Ethics,....................................... 184
Medical and Physical Science—Nashville Monthly Record of,.............................. 185
Medical and Surgical Reporter, Philadelphia, Penn.,........................................... 185
Medical Facts, &c.,................................. 189
Mammary Jacket—A Paper Read before the Montgomery Medical Society, Tenn., May 3d, 1851, by D. C. Higbee, M. D.,........ 206
Medical Skepticism—A few thoughts suggested by the present phase of,.............. 233
Medicine and Surgery—London Practice of, Metropolitan Free Hospital—Polycystic Ovarian tumor—Ovariotomy—Recovery, 246
Mammary Glands—Extraordinary Development of,............................................ 259
Mammary Abscess,................................. 270
Monstrosity—Singular Case of,.................. 300
Medical Facts,........................................ 319
Mammæ Inflamed, Ointment of Iodine and Belladonna in, by J. S. Weatherly, M. D., of Mongomery, Alabama,..................... 6
Maine Medical Association, Meeting of,..... 18
Maine Medical and Surgical Reporter,....... 64
Marriage, Influence of—on Mortality in France,............................................... 345
Meteorological Observations,................... 380
Meteorological Table,.............................. 388
Milk Sickness—its etiology, pathology, diagnosis and treatment,.......................... 414
Meteorological Table,.............................. 772
Medical News and Items,......................... 442
Meteorological Table,.............................. 458
Menstruation, Remarks upon, physiological and pathological, by Hayden Coe, M. D., Atlanta, Georgia,,.......................... 470
Medical Society of the State of Georgia,.... 505
Medical and Literary Weekly,................... 506
Medical and Literary Weekly, Prospectus,.. 506
Meteorological Table,.............................. 516
Medical Association of the State of Georgia—Abstract of the Proceedings of,...... 567
Means, M. D., Thos. A............................. 576
Medical Association of the State of Georgia, at its Annual Meeting, held in the City of Atlanta, April 13 and 14, 1859..... 581
Medical Association of the State of Georgia, Transactions of—Report of a Case of Vesico Vaginal Fistula, by Robert Battey, M. D.. of Rome, Georgia,........................ 591
Meteorology, Report on............................ 635
Meteorological Table for April,................. 643
Meteorological Table for May,.................. 644
Medical Association of Georgia, Transactions of—Remarks on the Pathology of Phlegmasia Dolens, by Hayden Coe, M. D., of Atlanta, Georgia,......................... 671
Medical and Literary Weekly,.................... 707
Masonic Signet and Journal,...................... 706
Meteorological Table,............................... 708

N

Nipples Inflamed during Lactaion,........... 172
News Items,........................................... 189
Navy Medical Service,.............................. 308
New Feature........................................... 314
Navy Medical Service............................... 317
News Items, &c.,.................................... 319
Nashville Journal—Infamous Attack upon Dr. S. D. Gross and the Jefferson Medical College, by Moxa,............................... 10
Nashville Journal,................................... 64
North Carolina Medical Journal,................ 64
New Arrangement,.................................. 376
New York Correspondence,....................... 763
New Medical Journals,............................. 379
New York Quarantine Difficulty,................ 510
New Orleans School of Medicine,.............. 635
Neuralgia Facei, by J. P. H. Brown, Dentist, Atlanta, Georgia,........................... 638
New York Correspondence,....................... 700

O

Opium, Use of in Children, by Thos. Pollard, M. D., Richmond, Va.,........................ 129
Our Journal,.......................................... 316
Obituary,.............................................. 318
Opium, Use of in certain conditions of the Parturient Process,............................. 614
Observations on Diphtheria, by Stephen Monckton, M. D.,................................ 744

P

Placenta Prævia—Case of Central Presentation—Safe Delivery,........................ 109
Puerperal Appoplectic Convulsions, Case of, with spontaneous expulsion of the fœtus after death,................................ 117
Pitting in Small-Pox, Prevention of, by T. S. Denny, M. D,, Atlanta, Ga.,............... 733
Professional Ollapodrida, by a Retired Pharmacien,............................................. 709
Payments for Jonrnal,.............................. 128
Publications Received,............................. 186
Professional Etiquette,............................. 187
Perineum, Laceration of, Prevention of,... 260
Prof. Physiology in Oglethorpe Medical College,............................................. 317
Poetry of Medicine, by J. S. Slaughter, Author of Madeline, &c., Atlanta, Ga.,......... 8
Payments, List of.................................... 387
Phthisis, Phosphorus in the Treatment of, 417
Pious Physician, Or, the Claims of Religion upon the Medical Profession,,.............. 419
Puerperal Diseases—Employment of Oil of Turpentine and Opium in large Doses, in the Treatment of Severe Cases,............. 504
Parisian Medicine and Surgery, Monthly Review of, by Theophilus Mack, M. D., Lecturer on Materia Medica and Therapeutics in the Medical Department of the University of Buffalo,........................... 757
Puerperal Phlebitis—Puerperal Infection, with Remarks,.................................... 539
Progeny—Comparative Influence of Male and Female Parents upon,..................... 557
Prolapsus Uteri, Case of, Cure without Operation, or, the Necessity of Wearing a Pessary,.............................................. 565

### INDEX TO FOURTH VOLUME MEDICAL & SURGICAL JOURNAL.

PAGE.

Parisian Medicine and Surgery, Monthly "Esquisse" of, 595
Professional Intercourse: A Valedictory Address to the Graduating Class of Shelby Medical College, Nashville, 604
Publications Received, 643
Puerperal Convulsions, Chloroform and Ether in, by S. W. Burney, M. D., Forsyth, 682
Preparatory Course in Atlanta Medical College, 699

**Q**

Quack Literature, Criminal and Obscene.... 322

Quackery in France, 449

**S**

"Southern Education for Southern Youth" 122
Spina Bifida, Case of, 235
Spina Bifida Successfully Treated by Ligature and Puncture, 236
Subscribers, 254
Subscriptions Received, 324
Suppuration of the Brain, by Wm. H. Phillpot, M. D., of Talbot co., Ga., 3
Specialities, 381
Small Pox at the Cape of Good Hope, 387
Spinal Column, remarks on some affections of, 408
Scarcity of Women in Victoria, 450
Southern Field and Fireside, 764
Strangulated Hernia Reduced by a Cold Douche—Translation—By F. H. Evans, M. D., of Aberdeen, Mississippi, 736
Snuff, Use of, 451
Subscriptions, 451
Sedative Application, 514
Subscriptions Received, 515
Spermatorrhœa: A Report by T. A. Means, M. D., of Memphis, Tenn., Read before the Medical Association of that city, 517

PAGE.

**T**

Types of Diseases and Therapy, Researches into, by Bennet Dowler, M. D., 137
Temperance, Georgia Crusader for 1859, 192
"Type, What is Change of in Disease," 230
Tumor Fibrous, of the Uterus Complicating Labor, 44

**U**

Uterus, 173
Uterine Hydatids, Case of, 211
Uterus, Displacement of, by J. G. Westmoreland, M. D., Atlanta, Ga., 348
University of the Pacific—Medical Department, 450
Utero Gestation—Second Impregnation at the Fourth Month of, 513
Umbilical Calculus, Report of a Case of, by S. H. Dean, M. D., of Conyers, Ga., 697
University of Louisville, 771

**V**

"Vix Medicatrix Naturæ," A Thesis on, Presented to the Faculty of Atlanta Medical College, by S. Watkins Vaughan, Jr., of Summerfield, Ala., for the Degree of M. D., Session 1858, Published by the Faculty, 262
Vesico-Vaginal Fistula, Two Cases of, Veratrum Viride, 54
Valedictory Address, 507
Variola, Application of Glycerine in, 580

**W**

Wine, Medical Uses of, 213

**Y**

Yellow Fever, 121

Yellow Fever, An Epistle on, 157

American Medical Association, ........

### EDITORIAL AND MISCELLANEOUS.

Atlanta Medical College—close of the Session, ........ 50

Veratrum Viride, ........ 54

Bibliographical, ........ 55

College Announcements Received, ........ 63

"Infamous Attack of the Nashville Journal, upon Dr. Gross and the Jefferson Medical College." ........ 64

The Medical Journal of North Carolina, ........ 64

Maine Medical and Surgical Reporter, ........ 64

Advertisements, ........ 64

---

### TO CORRESPONDENTS.

☞ All Communications pertaining to the Editorial Department of the Journal, should be addressed to the Editors.

☞ All Communications, having reference to the Subscription, the Advertising or the Financial Department, should be addressed to the Treasurer, J. G. WESTMORELAND, M. D., Dean of the Faculty of the Atlanta Medical College.

